# Acarbose Treatment and the Risk of Cardiovascular Disease in Type 2 Diabetic Patients: A Nationwide Seven-Year Follow-Up Study

**DOI:** 10.1155/2014/812628

**Published:** 2014-07-07

**Authors:** Jui-Ming Chen, Cheng-Wei Chang, Ying-Chieh Lin, Jorng-Tzong Horng, Wayne H.-H. Sheu

**Affiliations:** ^1^Department of Endocrinology and Metabolism, Tungs' Taichung MetroHarbor Hospital, Taichung 435, Taiwan; ^2^Department of Biomedical Informatics, Asia University, Taichung 413, Taiwan; ^3^Department of Gerontechnology and Service Management, Nan Kai University of Technology, Nantou 542, Taiwan; ^4^Department of Information Management, Hsing Wu University, New Taipei City 244, Taiwan; ^5^Department of Computer Science and Information Engineering, National Central University, Chungli 320, Taiwan; ^6^Division of Endocrinology and Metabolism, Department of Internal Medicine, Taichung Veterans General Hospital, 160, Section 3, Taichung-Harbor Road, Taichung 407, Taiwan; ^7^School of Medicine, National Defense Medical Center, Taipei 114, Taiwan; ^8^School of Medicine, National Yang-Ming University, Taipei 112, Taiwan; ^9^Institute of Medical Technology, National Chung-Hsing University, Taichung 402, Taiwan

## Abstract

*Objective*. To investigate the potential benefits of acarbose treatment on cardiovascular disease (CVD) in patients with type 2 diabetes by using nationwide insurance claim dataset. *Research Design and Methods*. Among 644,792 newly diagnosed type 2 diabetic patients without preexisting CVD in a nationwide cohort study, 109,139 (16.9%) who had received acarbose treatment were analyzed for CVD risk. Those with CVD followed by acarbose therapy were also subjected to analysis. *Result*. During 7 years of follow-up, 5,081 patients (4.7%) developed CVD. The crude hazard ratio (HR) and adjusted HR were 0.66 and 0.99, respectively. The adjusted HR of CVD was 1.19, 0.70, and 0.38 when the duration of acarbose use was <12 months, 12–24 months, and >24 months, respectively. Adjusted HR was 1.14, 0.64, and 0.41 with acarbose cumulative doses <54,750 mg, 54,751 to 109,500 mg, and >109,500 mg, respectively. *Conclusion*. In patients with type 2 diabetes without preexisting CVD, treatment with acarbose showed a transient increase in incidence of CVD in the initial 12 months followed by significant reductions of CVD in prolonged acarbose users. After the first CVD events, continuous use of acarbose revealed neutral effect within the first 12 months. The underlying mechanisms require further investigations.

## 1. Introduction 

Previous observational studies have indicated that postprandial hyperglycemia seems to play a unique role in the development of cardiovascular complications in patients with type 2 diabetes [1–4]. This is particularly relevant for Asian diabetic subjects whose postprandial hyperglycemia contributes more prominently to the level of HbA1c [5].

Acarbose, an oral antidiabetic agent, competitively inhibits the alpha-glucosidase in the brush-border of the small intestine and delays the digestion of complex carbohydrates in the upper small bowel that subsequently retards the absorption of glucose and blunts the postprandial hyperglycemia [6, 7]. Compared to metformin, acarbose is similar in efficacy to initial therapy in Chinese patients newly diagnosed with type 2 diabetes [8]. Results from the STOP NIDDM (Study to Prevent Non-Insulin-Dependent Diabetes Mellitus) had shown that acarbose treatment was associated with a 49% risk reduction in cardiovascular events in subjects with impaired glucose tolerance [9]. Even after adjusting for major risk factors, the reduction in the risk of cardiovascular events and hypertension associated with acarbose treatment was still statistically significant. In a meta-analysis of patients with type 2 diabetes, acarbose use was associated with a 35% reduction in cardiovascular events [10]. The mechanisms by which acarbose lowers the risk of cardiovascular events is putatively attributed to the diminution in postprandial hyperglycemic excursion and thus leads to a lowering of the impact of oxidative stress and tissues protection as well as a series of benefits [6, 11].

However, the above-mentioned findings were either limited to use in subjects with IGT (impaired glucose tolerance) [9], short-term studies [11], or meta-analysis [12]. Currently, there are no population-based long-term investigations about the potential cardiovascular effect of acarbose treatment in patients with type 2 diabetes. To address this issue, we used the Taiwanese National Health Insurance Research Database (NHIRD) to evaluate the cardiovascular effect of acarbose treatment in newly type 2 diabetic patients without previous CVD [13]. We also examined the effects of continuous use of acarbose on recurrent CVD in those diabetic subjects who had first cardiovascular events.

## 2. Research Design and Methods

### 2.1. Data Source

Since March 1, 1995, Taiwan had launched a single-payer National Health Insurance Program.

And in 2007, 98% of Taiwan's populations had been enrolled in this program [13]. Data in the National Health Insurance Research Database (NHIRD) that could be used to identify patients or care providers is scrambled before being sent for database construction. The database is further scrambled before being released to researchers [13].

### 2.2. Methods

In this study, diabetic patients were identified using the ICD-9-CM code (250) with at least 3 outpatient visits or hospitalization treatment once which has been validated. Diabetic subjects were enrolled if they fulfilled the following three criteria: (1) diagnosed with diabetes between year 2003 and 2008; (2) age older than 30 years and less than 100 years as of January 1, 2003; and (3) had no diagnosis of cardiovascular diseases before year 2003 or one year prior to the diagnosis of diabetes.

**Figure 1:**
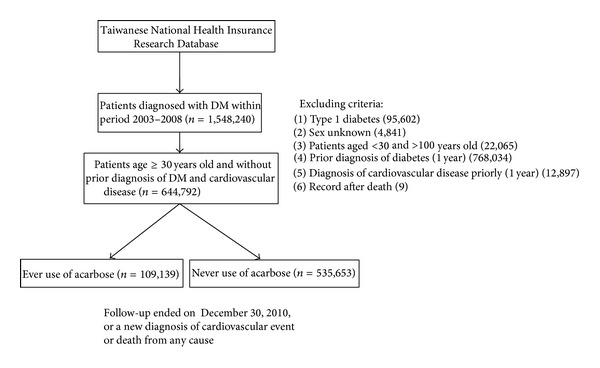
Study subjects' selection flow chart in those newly diagnosed with diabetes who do not have preexisting CVD.

Patients with type 1 diabetes, having diagnosis of diabetes in prior one year, and with sex unknown in the data were all excluded. The primary endpoint is cardiovascular diseases described below. The follow-up period started from Jan. 1, 2003 and lasted until Dec. 31, 2010 (Figure 1).

Cardiovascular outcomes were identified with the diagnosis of cardiovascular disease (coronary heart disease with ICD-9-CM codes 410–414, stroke with ICD-9CM codes 430–438, and peripheral arterial occlusive disease (PAOD) with ICD-9CM codes 443.9). To confirm the diagnosis, all above events required hospitalization for at least once.

The comorbidities, including hypertension (ICD-9CM codes 401–405), chronic kidney disease (CKD) (ICD-9CM codes 582-583), and hyperlipidemia (ICD-9CM codes 272), were recorded with at least 3 times of outpatient visit or admission once.

Using the NHIRD, a total of 644,792 newly diagnosed type 2 diabetic patients were enrolled. Among them 109,139 had received acarbose treatment during the period of follow-up. The follow-up period ended on December 30, 2010 or when a newly diagnosed cardiovascular event or death from any cause developed (Figure 1 and Table 1).

Cumulative duration of taking acarbose was calculated by counting the number of days between prescriptions. If the next prescription was filled within 30 days of the expected end date of the previous prescription, we assumed that therapy was uninterrupted. However, if there were no refills within the 30 days after the expected end date of the previous prescription, we assumed a gap in therapy starting 30 days after the date that the previous prescription should have ended. The cumulative duration variable was a time-varying sum of all periods of exposure even if there were gaps in treatment. Cumulative dose of acarbose was calculated in a similar fashion.

Among the 5081 patients who had received acarbose and developed CVD, 456 patients who died in the first month were excluded. A total of 4625 patients were enrolled in this study and followed up to record their recurrent CVD events. Among them, 1756 patients kept acarbose therapy while 2869 patients stopped receiving acarbose and switched to other antidiabetic regimens (see Supplemental Figure 1s of the Supplementary Material available online at http://dx.doi.org/10.1155/2014/812628).

The protocols had been submitted to the Tungs' Taichung MetroHarbor Hospital IRB (institutional review board) for review and approval was obtained.

### 2.3. Statistical Analysis

Distributions of subjects with and without use of acarbose according to age, gender, clinical comorbidities, and concomitant use of other diabetes medications were examined using *χ* ^2^-tests for categorical variables and Student's *t*-tests for continuous variables. The crude, age, gender, use of other diabetes medications, Charlson-Deyo comorbidity index (CCI), adjusted hazard ratio, and 95% confidence interval were calculated in subjects with and without use of acarbose, respectively. All analyses were performed using the SAS software, version 9.2 (SAS Institute, Cary, NC, USA).

## 3. Results

Among 644,792 patients with newly diagnosed type 2 diabetes identified during 2003 to 2008, 109,139 (16.9%) were treated with acarbose, either as monotherapy or in combination with other antidiabetic regimens. As compared with nonusers of acarbose, the acarbose users were younger, slightly female predominant, had more comorbidities, and were more likely to take combination treatment with other antidiabetic regimens. During 7 years (median 3.8 years) of follow-up, 5081 patients (4.7%) who had received acarbose treatment developed CVD as compared to 33,203 patients (6.2%) who never took acarbose therapy. Furthermore, 2619 patients (2.4%) who had received acarbose treatment developed stroke as compared to 18212 (3.4%) who never took acarbose therapy. The study showed that acarbose users group had a lower risk of developing CVD (*P* < 0.001) and stroke (*P* < 0.037) (Supplemental Table 1s). Table 1 depicted data of those with or without CVD in acarbose users. Those with CVD were older, male predominant, had more comorbidities with hypertension and CKD but were less likely to have hyperlipidemia (all *P* < 0.001), and had lesser use of combined oral hypoglycemic agents but more use of insulin as compared to those without CVD (Table 1).

By using those without exposure to acarbose as reference value, the crude (95% CI) hazard ratio (HR) of acarbose users who developed CVD was 0.66 (95% CI 0.641–0.680, *P* < 0.001) (Table 2). After adjusting for age, gender, Charlson-Deyo comorbidity index (CCI), and concomitant use of other antidiabetic regimens, the adjusted HR became 0.99 (0.958 to 1.019, *P* = 0.443). Further analysis indicated that adjusted HR of CVD was 1.19 (95% CI 1.152–1.231 *P* < 0.001), 0.70 (95% CI 0.643–0.762 *P* < 0.001), and 0.38 (95% CI 0.341–0.425 *P* < 0.001) when the duration of acarbose use to CVD events was <12 months, 12–24 months and >24 months, respectively. Adjusted HR was 1.14 (95% CI 1.105–1.18 *P* < 0.001), 0.64 (95% CI 0.583–0.704 *P* < 0.001), and 0.41 (95% CI 0.360-0.460 *P* < 0.001) with acarbose cumulative dose <54,750 mg, 54,751 to 109,500 mg, and >109,500 mg, respectively (Table 2).

For patients who had first event of CVD, continuous use of acarbose revealed neutral adjusted HR 1.00 (95% CI 0.891–1.132 *P* = 0.947) for recurrent CVD in those with use duration <12 months followed by lower adjusted HR in those with prolonged acarbose use as compared to those who stopped using acarbose (Table 3). Further analysis, based on cumulative dosages, showed a similar trend (Table 3).

## 4. Discussion 

The main findings from the present dataset indicated that use of acarbose, either as monotherapy or in combination with other antidiabetic regimens, in newly diagnosed type 2 diabetes subjects who did not have preexisting CVD, provided a unique impact on the subsequent development of CVD. Specifically, use of acarbose increased the chances of developing CVD in the first 12 months of exposure. However, the benefits on CVD began to emerge in continuous users of acarbose. Analysis of dosages of acarbose showed similar patterns.

Results from previous observational studies have demonstrated that elevated postprandial glycemic excursion contributes to increase in the risk of developing coronary heart disease or a CV event [14, 15]. By reducing postprandial hyperglycemia, the potential benefits derived from acarbose treatment have included reduced body weight [9, 12], reduced blood pressure both systolic and diastolic [9, 10, 16, 17], reduced triglyceride [18], reduced post meal lipemia [19–21], reduced postmeal activation of coagulation [22], reduced insulin resistance [18, 23], reduced carotid intima-medial thickness [24], reduced myocardial infarct size [25], marked decrease in the plasma levels of plasminogen activator inhibitor 1 and fibrinogen, and reduction in the plasma levels of oxidized low-density lipoprotein [26]. In fact, findings from STOP NIDDM [9] in subjects with IGT and results from MeRIA study [12] all support the potential benefits of acarbose management on CVD.

The exact causes of our unanticipated findings that acarbose increased CVD in the first 12 months of exposure while providing protection in those prolonged users are not clear at this moment. Our target diabetic subjects were newly diagnosed with diabetes without preexisting CVD which was different from the subjects of STOP NIDDM [1] and MeRIA [12]. Considering the time needed to form atherosclerosis, it is not reasonable to speculate that the use of acarbose enhanced occurrence of CVD events. In this regard, there has also no evidence that acarbose could precipitate rupture of those preexisting vascular plaques in acarbose users. Actually, recent study indicated that the use of acarbose could rapidly improve carotid plaque echolucency within 1 month of therapy in type 2 diabetes patients with acute coronary syndrome [27]. On the other hand, diabetic subjects who used acarbose for a prolonged period or who reached certain amount of doses showed benefits of protection, both in first CVD or recurrent CVD events. The exact mechanisms definitely require further investigations.

There are several limitations in our study. First, a causal association between acarbose and CVD cannot be ascertained based on the observational data. We cannot retrieve clinical data like lipids and glycemic, blood pressure control from this claim dataset. Although we have adjusted potential confounding, there may be other measured and unmeasured factors which we were not able to detect or obtain. The diagnosis of type 2 diabetes or the cardiovascular events according to the ICD-9-CM may also lead to some distortion.

## 5. Conclusions

Our study suggests that treatment with acarbose in the newly diagnosed type 2 diabetic patients who do not have preexisting CVD showed an initial increased HR followed by benefits on subsequent CVD. After the first CVD events, continuous use of acarbose revealed a relatively similar pattern of benefits on developing recurrent CVD. The effects of acarbose on CVD remain speculative before the final results of other large prospective studies come out (such as the ongoing Acarbose Cardiovascular Evaluation Trial) [28].

## Supplementary Material

1, Demographics, Disease and Treatment Characteristics of those treated with or without Acarbose; 2, flow chart of the sub-study about recurrence of cardiovascular disease.

## Figures and Tables

**Table 1:** Demographics, comorbidities, and concomitant use of antidiabetic medications in those acarbose users with (*n* = 5081) or without (104058) CVD development.

Characteristic	Overall number	Did not develop CVD	Developed CVD	*P* value
	109,139	104,058	5,081	
Age, years (mean ± SD)				
30–39	9,417 (8.6)	9,239 (8.9)	178 (3.5)	<0.0001
40–49	25,707 (23.6)	24,936 (24.0)	771 (15.2)	
50–59	32,921 (30.2)	31,496 (30.3)	1,425 (28.0)	
60–69	22,343 (20.5)	21,035 (20.2)	1,308 (25.7)	
70–79	14,295 (13.1)	13,269 (12.8)	1,026 (20.2)	
80–89	4,152 (3.8)	3,805 (3.7)	347 (6.8)	
>=90	304 (0.3)	278 (0.3)	26 (0.5)	
Sex				
Male	59,473 (54.5)	56,033 (53.8)	3,440 (67.7)	<0.0001
Female	49,666 (45.5)	48,025 (46.2)	1,641 (32.3)	
Comorbidities				
Hypertension	81,341 (74.5)	76,944 (73.9)	4,397 (86.5)	<0.0001
Hyperlipidemia	70,106 (64.2)	67,213 (64.6)	2,893 (56.9)	<0.0001
CKD	6,965 (6.4)	6,348 (6.1)	617 (12.1)	<0.0001
Other diabetes medications				
Rosiglitazone	9,420 (8.6)	9,010 (8.7)	410 (8.0)	<0.0001
Metformin	72,378 (66.3)	70,037 (67.3)	2,341 (46.1)	<0.0001
Pioglitazone	14,963 (13.7)	14,557 (14.0)	406 (8.0)	<0.0001
Sulfonylurea	51,765 (47.4)	49,915 (48.0)	1,850 (36.4)	<0.0001
Meglitinide	13,161 (12.1)	12,551 (12.1)	610 (12.0)	<0.0001
Insulin	8,994 (8.2)	8,426 (8.1)	568 (11.2)	<0.0001
DPP4 inhibitor	11,641 (10.7)	11,528 (11.1)	113 (2.2)	<0.0001

CVD: cardiovascular disease. DPP4: dipeptidyl peptidase 4. CKD: chronic kidney disease.

**Table 2:** Crude and adjusted hazard ratio of CVD with or without acarbose use.

Risk factor	Developed CVD	Did not develop CVD	Crude				Adjusted			
			HR	95% CI		*P*	HR	95% CI		*P*
				Low	Up			Low	Up	
Never use of acarbose	33203	502450	Ref.	—	—		Ref.	—	—	—
Ever use of acarbose	5081	104058	0.66	0.641	0.680	<0.001	0.99	0.958	1.019	0.443
Cumulative duration of therapy (months)										
<12	4216	69976	0.83	0.802	0.855	<0.001	1.19	1.152	1.231	<0.001
12–24	545	17345	0.42	0.387	0.459	<0.001	0.70	0.643	0.762	<0.001
>24	320	16737	0.24	0.217	0.271	<0.001	0.38	0.341	0.425	<0.001
Cumulative dose (mg)										
1–54,750	4373	75757	0.79	0.769	0.819	<0.001	1.14	1.105	1.18	<0.001
54,751–109,500	449	15273	0.39	0.357	0.43	<0.001	0.64	0.583	0.704	<0.001
>109,500	259	13028	0.25	0.219	0.28	<0.001	0.41	0.360	0.460	<0.001

Adjusted age, gender, other diabetic medications, and CCI. CVD: cardiovascular disease.

**Table 3:** HR of recurrent CVD by keep or stop using acarbose.

Risk factor	Recurrence of CVD	No recurrence of CVD	Crude				Adjusted			
			HR	95% CI		*P*	HR	95% CI		*P*
				Low	Up			Low	Up	
Stop acarbose users	627	2242	Ref.	—	—		Ref.	—	—	—
Keep acarbose users	582	1174	0.84	0.752	0.944	0.003	0.88	0.781	0.987	0.029
Cumulative duration of therapy (months)										
<12	508	1142	0.98	0.87	1.1	0.715	1.00	0.891	1.132	0.947
12–24	49	18	0.52	0.392	0.702	<0.001	0.48	0.355	0.645	<0.001
>24	25	14	0.29	0.196	0.441	<0.001	0.38	0.249	0.567	<0.001
Cumulative dose (mg)										
1–10500	523	1149	0.95	0.848	1.07	0.413	0.97	0.86	1.091	0.600
10501–28000	39	16	0.45	0.324	0.621	<0.001	0.47	0.338	0.657	<0.001
>28000	20	9	0.33	0.211	0.518	<0.001	0.40	0.253	0.628	<0.001

Adjusted age, gender, other diabetic medications, and CCI. CVD: cardiovascular disease.
