# Supplementary material for: Acarbose Treatment and the Risk of Cardiovascular Disease in Type 2 Diabetic Patients: A Nationwide Seven-Year Follow-Up Study
Source: J Diabetes Res. 2014 Jul 7;2014:812628. doi: 10.1155/2014/812628 (PMC4147291; doi:10.1155/2014/812628)
Supplement: Supplementary file 1 — 1, Demographics, Disease and Treatment Characteristics of those treated with or without Acarbose; 2, flow chart of the sub-study about recurrence of cardiovascular disease. [file 812628.f1.zip › 979320.docx]

| Supplemental Table 1s. Demographics, Disease and Treatment Characteristics of those treated with (n=109,139) or without (n=535,653) Acarbose | | | | | |
| --- | --- | --- | --- | --- | --- |
| Characteristics | | No. Overall | Treated by Acarbose | Never treated by Acarbose |  |
|  |  | 644,792 | 109,139 | 535,653 | P value |
| Age, years(mean ± SD) |  |  |  |  |  |
|  | 30-39 | 47,780(7.4) | 9,417(8.6) | 38,363(7.2) | <.0001 |
|  | 40-49 | 128,891(20.0) | 25,707(23.6) | 103,184(19.3) |  |
|  | 50-59 | 183,590(28.5) | 32,921(30.2) | 150,669(28.1) |  |
|  | 60-69 | 134,412(20.8) | 22,343(20.5) | 112,069(20.9) |  |
|  | 70-79 | 102,145(15.8) | 14,295(13.1) | 87,850(16.4) |  |
|  | 80-89 | 42,213(6.5) | 4,152(3.8) | 38,061(7.1) |  |
|  | >=90 | 5,761(0.9) | 304(0.3) | 5,457(1.0) |  |
| Sex | |  |  |  |  |
|  | Male | 354,792(55.0) | 59,473(54.5) | 295,319(55.1) | 0.0001 |
|  | Female | 290,000(45.0) | 49,666(45.5) | 240,334(44.9) |  |
| Comorbidities |  |  |  |  |  |
|  | Hypertension | 447,081(69.3) | 81,341(74.5) | 365,740(68.3) | <.0001 |
|  | Hyperlipidemia | 336,298(52.2) | 70,106(64.2) | 266,192(49.7) | <.0001 |
|  | CKD | 31,374(4.9) | 6,965(6.4) | 24,409(4.6) | <.0001 |
| Other diabetes medications |  |  |  |  |  |
|  | Rosiglitazone | 22,023(3.4) | 9,420(8.6) | 12,603(2.4) | <.0001 |
|  | Metformin | 285,328(44.3) | 72,378(66.3) | 212,950(39.8) | <.0001 |
|  | Pioglitazone | 36,498(5.7) | 14,963(13.7) | 21,535(4.0) | <.0001 |
|  | Sulfonylurea | 188,097(29.2) | 51,765(47.4) | 136,332(25.5) | <.0001 |
|  | Meglitinide | 34,017(5.3) | 13,161(12.1) | 20,856(3.9) | <.0001 |
|  | Insulin | 24,184(3.8) | 8,994(8.2) | 15,190(2.8) | <.0001 |
|  | DPP4 inhibitor | 25,886(4.0) | 11,641(10.7) | 14,245(2.7) | <.0001 |
| Event |  |  |  |  |  |
|  | Cardiovascular | 38,284 | 5,081(4.7) | 33,203(6.2) | <.001 |
|  | Stroke | 20,831 | 2,619(2.4) | 18,212(3.4) | <.037 |
|  | Death | 79,894 | 9,027(8.3) | 70,867(13.2) | <.0001 |

Abbreviation: CKD: chronic kidney disease, DPP4: Dipeptidyl peptidase-4
