# Supplementary material for: Acarbose Treatment and the Risk of Cardiovascular Disease in Type 2 Diabetic Patients: A Nationwide Seven-Year Follow-Up Study
Source: J Diabetes Res. 2014 Jul 7;2014:812628. doi: 10.1155/2014/812628 (PMC4147291; doi:10.1155/2014/812628)
Supplement: Supplementary file 1 — 1, Demographics, Disease and Treatment Characteristics of those treated with or without Acarbose; 2, flow chart of the sub-study about recurrence of cardiovascular disease. [file 812628.f1.zip › 979316.doc]

Received acarbose and developed CVD (n=5081)

Received acarbose , developed CVD

Survived >= 1 month (n=4625)

Excluding:

1. Death in the first month (n=456)

keep using acarbose (n=1756)

Did not keep using acarbose (n=2869)

Follow-up ended on 30 December 2010,

Or or a cardiovascular event

or or death from any cause

Supplemental Fig 1s
